# Advanced Estimation of Winter Wheat Leaf’s Relative Chlorophyll Content Across Growth Stages Using Satellite-Derived Texture Indices in a Region with Various Sowing Dates

**DOI:** 10.3390/plants14152297

**Published:** 2025-07-25

**Authors:** Jingyun Chen, Quan Yin, Jianjun Wang, Weilong Li, Zhi Ding, Pei Sun Loh, Guisheng Zhou, Zhongyang Huo

**Affiliations:** 1 Jiangsu Key Laboratory of Crop Genetics and Physiology/Jiangsu Key Laboratory of Crop Cultivation and Physiology, Agricultural College, Yangzhou University, Yangzhou 225009, China; 231704103@stu.yzu.edu.cn (J.C.); mx120220725@stu.yzu.edu.cn (Q.Y.); mx120220735@stu.yzu.edu.cn (W.L.); mz120241392@stu.yzu.edu.cn (Z.D.); huozy69@163.com (Z.H.); 2 Jiangsu Co-Innovation Center for Modern Production Technology of Grain Crops, Yangzhou University, Yangzhou 225009, China; 3 Institute of Marine Geology and Resources, Ocean College, Zhejiang University, Zhoushan 316021, China; psloh@zju.edu.cn; 4 Joint International Research Laboratory of Agriculture and Agricultural Product Safety, Yangzhou 225009, China; gszhou@yzu.edu.cn

**Keywords:** precision agriculture, Sentinel-2, winter wheat, SPAD value, texture indices, machine learning

## Abstract

Accurately estimating leaves’ relative chlorophyll contents (widely represented by Soil and Plant Analysis Development (SPAD) values) across growth stages is crucial for assessing crop health, particularly in regions characterized by varying sowing dates. Unlike previous studies focusing on high-resolution UAV imagery or specific growth stages, this research incorporates satellite-derived texture indices (TIs) into a SPAD value estimation model applicable across multiple growth stages (from tillering to grain-filling). Field experiments were conducted in Jiangsu Province, China, where winter wheat sowing dates varied significantly from field to field. Sentinel-2 imagery was employed to extract vegetation indices (VIs) and TIs. Following a two-step variable selection method, Random Forest (RF)-LassoCV, five machine learning algorithms were applied to develop estimation models. The newly developed model (SVR-RBF_VIs+TIs_) exhibited robust estimation performance (*R*^2^ = 0.8131, *RMSE* = 3.2333, *RRMSE* = 0.0710, and *RPD* = 2.3424) when validated against independent SPAD value datasets collected from fields with varying sowing dates. Moreover, this optimal model also exhibited a notable level of transferability at another location with different sowing times, wheat varieties, and soil types from the modeling area. In addition, this research revealed that despite the lower resolution of satellite imagery compared to UAV imagery, the incorporation of TIs significantly improved estimation accuracies compared to the sole use of VIs typical in previous studies.

## 1. Introduction

Leaves’ relative chlorophyll content (widely represented by Soil and Plant Analysis Development (SPAD) values), as a key physiological parameter reflecting photosynthetic efficiency, growth status, and the nutritional level of crops, has been widely employed in the assessment of crop health [1,2,3]. In recent years, accumulating research has confirmed that remote sensing (RS) technology possesses significant advantages—such as non-invasiveness and high efficiency—in monitoring crops’ SPAD values, making it an ideal tool for this purpose [4,5]. Although unmanned aerial vehicle (UAV)-based RS has advanced rapidly in the context of high-resolution monitoring, satellite-based RS continues to play a dominant role in field-scale or large-area crop monitoring applications [6].

In Jiangsu Province, China, the agricultural production structure is predominantly characterized by small-scale farmers, with relatively few large-scale agricultural operations. At present, small family farms with an average cultivated area of less than one hectare remain the dominant form of agricultural management in the region [7]. As a consequence, the sowing dates of winter wheat exhibit considerable variation across the area [8]. These variations in sowing time significantly influence the growth and development of winter wheat by altering the duration of sunlight and the accumulation of temperature during different phenological stages. This leads to the asynchronous growth stages of winter wheat in different fields at the same point in time [9].

Previous RS-based studies have primarily focused on developing SPAD value estimation models for winter wheat during specific growth stages under uniform sowing dates [10,11]. While these models may exhibit high estimation accuracy under certain conditions, numerous studies have indicated that their applicability is limited during other growth stages, and that their estimations are often unstable, lacking broad generalizability [12,13,14]. Although these models have shown success in controlled environments, their practical application is significantly constrained in real-world agricultural production, particularly in scenarios with diverse sowing dates. This issue is particularly pronounced in Jiangsu Province, China, where most agricultural fields are managed by small-scale farmers, leading to multiple sowing dates for winter wheat.

Furthermore, crop parameter estimation models developed using data from the entire growth cycle demonstrate more stable performance compared to models based solely on data from specific growth stages [15]. This stability stems from the model’s ability to compensate for discrepancies observed at particular growth stages by integrating observations from other stages [13,16]. Dhillon et al. [17] further assert that employing time series RS imagery yields more reliable and accurate estimates of winter wheat growth parameters than relying on a single image from a specific growth stage.

The estimation of SPAD values generally relies on physical modeling approaches (i.e., the PROSAIL model), empirical modeling, or hybrid methods that combine both. However, numerous parameters in physical models are difficult to obtain, which limits their practical application in crop parameter estimation. While hybrid methods can improve estimation accuracy, their instability remains a critical issue due to the challenge of balancing the interface between inversion algorithms and physical models. In contrast, empirical modeling typically combines vegetation indices (VIs) with machine learning techniques to develop regression models. This approach is preferred for SPAD value estimation due to its stability and higher estimation reliability. However, solely relying on VIs significantly limits the development of a comprehensive SPAD value estimation model for winter wheat throughout its entire growth cycle.

The main issue lies in the fact that when VIs are used for modeling SPAD values, saturation problems often occur, especially in environments with high vegetation cover [18]. Additionally, it is noteworthy that during the grain-filling period, despite lower SPAD values due to the transfer and transport of assimilates, the top spike of the winter wheat canopy remains green 14. The presence of both leaves and spikes in the winter wheat canopy makes the reflectance signal more complex, which introduces significant uncertainty when measuring chlorophyll levels in the canopy’s reflectance spectra. Numerous studies have shown that although VIs exhibit a strong correlation with crop parameters at specific growth stages, they are difficult to extrapolate to other growth stages [19,20]. Consequently, these factors present significant challenges in developing a cross-growth-stage SPAD value estimation model that is applicable throughout the entire growth cycle of winter wheat.

Fortunately, by leveraging the spatial variations in pixel intensity, extracting texture indices (TIs) from RS images can effectively reveal crop canopy structure and geometric features, thereby alleviating this challenge to some extent [21]. For instance, Guo et al. [22] significantly improved the estimation accuracy of maize SPAD values by combining VIs and TIs extracted from multispectral UAV images. However, an extensive literature review reveals that most existing studies have focused on extracting TIs from high-resolution UAV-based RS images, with limited attempts to use texture indices derived from satellite RS images to estimate crop parameters. This disparity arises from the significantly lower resolution of satellite RS imagery compared to UAV imagery, leading previous studies to generally perceive accurate crop parameter estimation from satellite RS images as a major challenge [14].

This study aimed to develop a satellite imagery-based model for the accurate estimation of SPAD values that incorporates multiple growth stages of winter wheat. We hypothesized that the capability of satellite-derived TIs enabled this model to be effectively applied in regions with temporal variations in sowing dates. This research represents the first attempt to estimate winter wheat SPAD values using TIs extracted from satellite RS imagery.

## 2. Materials and Methods

### 2.1. Study Area

**Figure 1:**
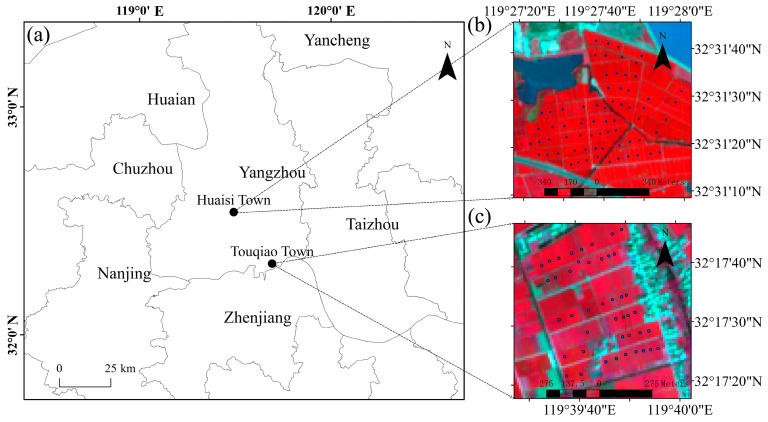
Geographic location and sampling site distribution of the study area. (**a**) The geographical locations of the sub-study areas of Huaisi Town and Touqiao Town; (**b**) the spatial distribution of sampling sites in Huaisi Town, overlaying a false-color composite image (Bands 8, 4, and 3 of Sentinel-2A) acquired on 11 April 2023; and (**c**) the spatial distribution of sampling sites in Touqiao Town, overlaying a false-color composite image (Bands 8, 4, and 3 of Sentinel-2A) acquired on 8 April 2023.

The research was carried out from 2022 to 2023. As depicted in Figure 1, the study area consists of two sub-study areas, namely Huaisi Town (32°31′18″ N, 119°28′6″ E) and Touqiao Town (32°17′31″ N, 119°39′37″ E), both located in Yangzhou City, Jiangsu Province, China.

It is particularly noteworthy that the measured fields in Huaisi Town in this research are cultivated by individual smallholder farmers, with significant variations in fertilization and irrigation practices. The soil type of the fields is sandy loam soil [23]. Conversely, the measured fields in Touqiao Town are cultivated uniformly by much bigger farm owners, with consistent fertilization and irrigation management practices. The soil type of the fields is mucky soil.

These two sub-study areas are located in the mid-lower Yangtze River area and predominantly employ a rice–wheat rotation system. For a long time, the sowing time for winter wheat in the mid-lower Yangtze River area has been affected by tight rice stubble, often resulting in uncertainty [24]. Upon on-site investigation, it was observed that the growth of winter wheat in Huaisi Town (with the variety Yangmai 29) and Touqiao Town (with the variety Zhenmai 18) is not synchronized, particularly evident from significant differences during the vegetative growth stage of winter wheat. For instance, in general, the wheat crops in Huaisi Town and Touqiao Town were observed to be at the heading stage and jointing stage, respectively, on April 10, 2023. Furthermore, the total sowing period in Huaisi Town was stratified into three phenologically distinct phases (normal, intermediate, and late), with inter-phase intervals averaging around 20 days, despite the predominance of winter wheat cultivation occurring during the conventional sowing window.

### 2.2. Data Collection

#### 2.2.1. Measured SPAD Values for Winter Wheat

In the sub-study area of Huaisi Town, the measured SPAD values of winter wheat were obtained at the growth stages of tillering, green-up, heading, and grain-filling. In the sub-study area of Touqiao Town, the measured SPAD values were obtained at the growth stages of jointing and grain-filling.

Typically, the measurement of SPAD values was conducted on the uppermost fully expanded leaves at different growth stages, as they represented significant variations in SPAD values among individual plants [25,26]. In the vegetative growth stage of winter wheat (tillering, green-up, and jointing), SPAD values offered crucial insights into the crop’s nutritional status, facilitating timely nitrogen fertilizer supplementation. During the reproductive growth stage (heading and grain-filling stages), SPAD values provided highly precise forecasts of yield [27].

At each sampling site, which was 20 m long and 20 m wide, 25 winter wheat plants were randomly chosen for actual SPAD value measurements, employing the non-destructive and portable SPAD-502plus handheld chlorophyll meter (Minolta Camera Co.; Osaka, Japan). In the initial stages of tillering, green-up, and jointing, data were collected from the top, central, and bottom sections of the second-to-last leaf (i.e., the uppermost fully expanded leaf) of the selected plants. During the heading and grain-filling stages, the flag leaf was measured. The mean of these measurements was used as the measured SPAD value for each selected plant. The SPAD value for each specific field was determined by averaging the actual SPAD value measurements from the 25 selected wheat plants.

#### 2.2.2. Acquisition of Sentinel-2 Images

Field-scale crop monitoring often necessitates high-resolution optical imagery to discern and extract complex crop growth characteristics [28]. It is noteworthy that each sampling plot in this study measured 20 m × 20 m, within which 25 winter wheat plants were randomly selected for in situ SPAD value measurements. Sentinel-2 MSI imagery—with native spatial resolutions of 10 m, 20 m, and 60 m across various bands—was resampled to a consistent 10 m-resolution reflectance dataset. This spatial scale is well aligned with the field plot dimensions, ensuring that each 10 m pixel integrates spectral signals from a physiologically meaningful portion of the canopy. Hence, it provides sufficiently detailed spectral and structural information to estimate SPAD values at the field level [29].

Sentinel-2 MSI imagery data of winter wheat during its crucial growth stages was obtained using the Google Earth Engine (GEE) platform. This platform has given us access to a wide range of satellite RS tools, including Level-1C and Level-2A products from Sentinel-2. The Level-2A product is generated by performing atmospheric correction on the Level-1C product and represents the Surface Reflectance [30]. The frequent revisit rate of Sentinel-2 of every five days [31] allows for the acquisition of at least one cloud-free image during each critical growth stage of winter wheat. This frequent revisit rate significantly enhances the practicality of our research methodology. For this research, the Level-2A product was chosen for subsequent analysis. Table 1 comprehensively lists the specific Sentinel-2 optical satellite imagery data utilized in this research. It is worth noting that the field data collection and model development were initially conducted in Huaisi Town. To further assess the robustness and spatial transferability of the developed model, Touqiao Town was subsequently incorporated as an independent validation site. Although only two cloud-free Sentinel-2 images were available for Touqiao Town, these acquisitions coincided with key growth stages of winter wheat, thereby enabling a representative and meaningful evaluation of model performance.

### 2.3. Extraction and Construction of RS Variables

Subsequently, all band data of the sampling points were extracted on the Google Earth Engine (GEE) platform (https://earthengine.google.com/ (accessed on 30 June 2024)), including B1, B2, B3, B4, B5, B6, B7, B8, B8A, B9, B11, and B12, and were resampled to 10 m reflectance data. Subsequently, they were used to calculate the VIs used for estimating SPAD values (Table 2).

The Gray-Level Co-occurrence Matrix (GLCM) initially proposed by Haralick in 1973 [52] is a prominent technique for texture analysis. Its popularity stems from variables such as rotational invariance, the capability to operate at multiple scales, and computational efficiency [53]. Acknowledging the influence of spatial resolution on TIs, GLCM-TIs were extracted from single-band images (B2, B3, B4, and B8) at a 10 m resolution on the GEE platform. The glcmTexture function was applied with a 3 × 3 moving window, and the results were averaged across four principal directions (0°, 45°, 90°, and 135°). The TIs used for estimating SPAD values in winter wheat are enumerated in Table 2.

To ensure the comprehensiveness of the input variables, a total of 96 RS variables were constructed, comprising both spectral and spatial information. Specifically, 36 VIs were derived from 12 spectral bands of Sentinel-2 MSI (Level-2A) imagery and their pairwise combinations. Additionally, 60 TIs were extracted from four 10 m resolution bands (B2, B3, B4, and B8) using 15 GLCM-based metrics, thereby capturing the spatial heterogeneity and structural characteristics of the crop canopy.

### 2.4. Variable Selection Method

Owing to the high-dimensional nature of the feature space (96 RS variables in total), rigorous variable selection must be implemented as a critical preliminary step in the modeling pipeline. However, employing a single variable selection method alone, like most previous studies did, may not effectively identify the most critical variables [54]. By addressing the issue of redundant RS image variables, previous studies have shown that a two-step variable selection process can markedly reduce the variable set without compromising the model’s accuracy, and even enhance the model’s performance [55,56]. Therefore, in this research, a two-step variable selection method, RF-LassoCV (initially using Random Forest for preliminary variable selection followed by LassoCV), was used, with the expectation of reducing data redundancy, optimizing the model, and producing reliable results with high computational efficiency. Variable selection was combined with five-fold cross-validation (CV) in this research.

### 2.5. Machine Learning Regression Models

Our primary objective in this research was to develop a strong model for accurately estimating the SPAD values of winter wheat from the tillering stage to the grain-filling stage. To achieve this, various machine learning regression models were explored, including RF [57], Support Vector Regression (SVR) [58] with different kernels (SVR-RBF, SVR-Poly, SVR-Sigmoid, and SVR-Linear), CatBoost [59], Backpropagation Neural Network (BPNN) [60], Long Short-Term Memory (LSTM) [61], and ElasticNet [62].

The parameters and non-parameters of these machine learning models were optimized using CV and grid search algorithms. By comparing the performance of these models, the most suitable model for winter wheat SPAD value estimation could be selected.

**Figure 2:**
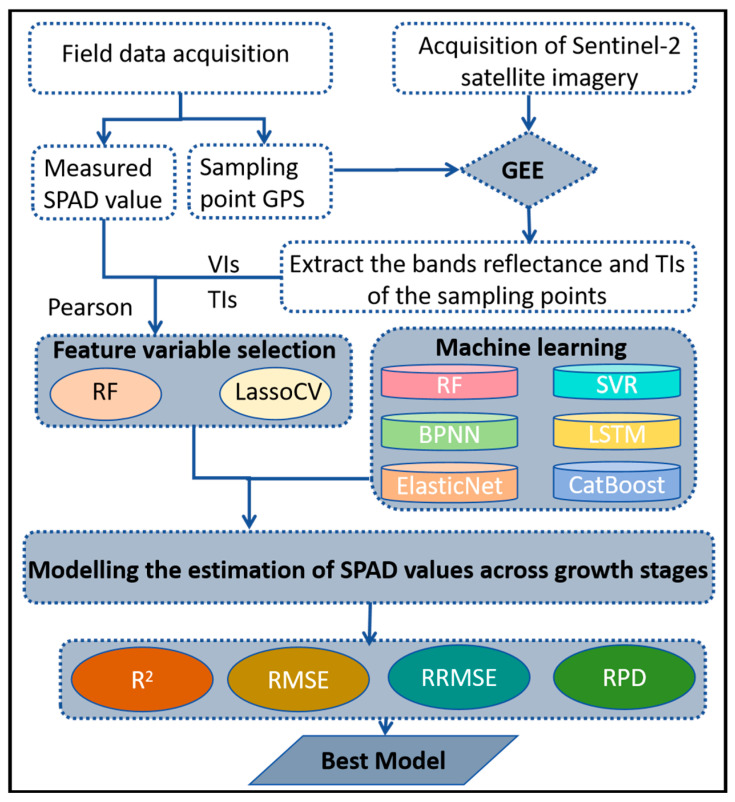
Schematic diagram of the optimal model’s development.

The specific modeling procedure is illustrated in Figure 2. To interpret the optimal model behavior and evaluate the contribution of each variable to the estimation of the target output (response variable), Shapley Additive Explanations (SHAP) analysis, as proposed by Lundberg and Lee [63], was utilized. It is important to emphasize that SHAP values do not infer causality; instead, they provide insights into the optimal model’s behavior in relation to the estimation outcomes.

In this research, the dataset of Huaisi Town was split into a training dataset and a testing dataset, adhering to a ratio of 8:2, through a randomized division process. In order to enhance the model’s performance and prevent overfitting, a K-fold CV approach (K = 5) was utilized during the training phase (with a sample size of 167). The models’ performance was subsequently assessed using the separate testing dataset that was not utilized during the model’s development process.

To rigorously evaluate the spatiotemporal transferability of the optimal model developed from the Huaisi Town training dataset, this research employed the Touqiao Town sub-study area dataset as an independent validation set. Notably, these two sub-study areas exhibited distinct agricultural phenological characteristics, particularly in sowing dates. Furthermore, the Touqiao Town dataset encompassed the critical jointing growth stage, a phenological phase absent in the Huaisi Town dataset. These substantive differences significantly strengthen the robustness of our validation approach for assessing the model’s spatiotemporal transferability within the region.

### 2.6. Model Performance Evaluation Metrics

For the evaluation of the model’s accuracy, our focus was directed toward four pivotal metrics: the Coefficient of Determination (R^2^, explicated in Equation (1)), Root Mean Square Error (RMSE, explicated in Equation (2)), Relative RMSE (RRMSE, explicated in Equation (3)), and the Ratio of Percentage Deviation (RPD, explicated in Equation (4)). Ordinarily, a higher R^2^ value, complemented by lower RMSE and RRMSE values, signifies an enhanced model performance. When interpreting RPD values in the article by Viscarra Rossel et al. [64], a value below 1.4 indicates very poor or poor estimations, 1.4 ≤ RPD < 1.8 suggests fair estimations, 1.8 ≤ RPD < 2.0 signifies good estimations, 2.0 ≤ RPD < 2.5 points to very good estimations, and RPD ≥ 2.5 denotes excellent estimations.

(1) $$R^{2}=\frac{\sum {\left({\hat{y}}_{i}-\bar{y}\right)}^{2}}{\sum {\left(y_{i}-\bar{y}\right)}^{2}}$$

(2) $$RMSE=\sqrt{\frac{\sum_{i=1}^{n}{\left(\hat{y_{i}}-y_{i}\right)}^{2}}{n}}$$

(3) $$RRMSE=\frac{RMSE}{\bar{y}}$$

(4) $$RPD=\frac{SD}{RMSE}$$

where $y_{i}$ is the measured SPAD value of sample *i*; ${\hat{y}}_{i}$ is the estimated SPAD value of sample i; $\bar{y}$ is the mean SPAD value; and n is the number of samples. SD is the standard deviation between the estimated and measured SPAD values.

## 3. Results

### 3.1. Statistical Analysis of Measured SPAD Values

Table 3 presents comprehensive observations of winter wheat SPAD values at various growth stages in the two sub-study areas of Huaisi Town and Touqiao Town. Notably, the cumulative SPAD value analysis across all growth stages in Huaisi Town yielded a coefficient of variation (C·V) of 17.41%, emphasizing temporal heterogeneity. According to Kahaer and Tashpolat [65], a C·V < 15% indicates a slight variation, while 15% < C·V < 36% denotes a moderate variation. A higher C·V is beneficial for the following development of models, as it signifies improved applicability and robustness.

### 3.2. Variable Selection

**Figure 3:**
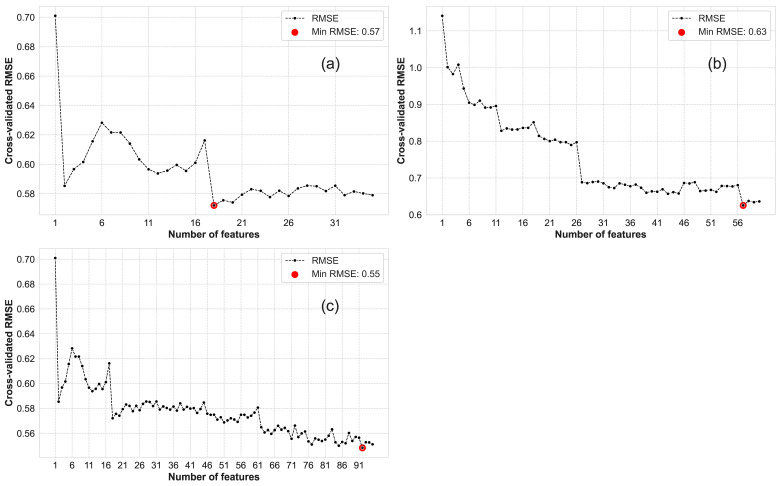
RF variable selection curve. (**a**) VIs; (**b**) TIs; and (**c**) VIs+TIs.

A two-step approach was adopted, initially utilizing RF for variable selection followed by LassoCV, to identify the optimal combination of RS variables for subsequent modeling. The RF variable selection process revealed that the RF method appeared to have limitations in effectively discerning the optimal variables for further modeling. The RF variable selection curve (Figure 3) serves to identify the minimum number of variables that provide optimal modeling capability (determined through 5-fold CV). The set of variables corresponding to the minimum *RMSE* represents the optimal variables. Within the set of VIs, a total of 18 variables were identified as optimal. In the case of the TI variable set, a total of 57 variables were identified as optimal. Furthermore, in the VI+TI variable set, a total of 92 variables were identified as optimal. Relying solely on RF variable selection in this research fails to effectively identify the most critical variables or substantially reduce the variable count.

After performing RF variable selection, LassoCV was further employed for variable selection, aiming to refine the variable set by penalizing the absolute size of the regression coefficients (alpha = 0.01). This approach is anticipated to yield a more streamlined and potentially more effective set of variables for estimation modeling.

**Figure 4:**
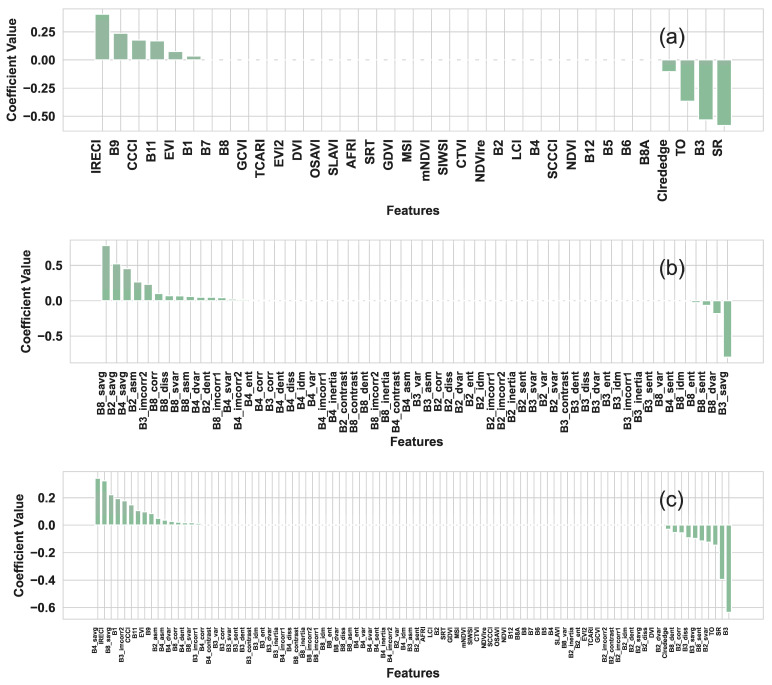
Variable importance ranking from LassoCV variable selection. (**a**) VIs; (**b**) TIs; and (**c**) VIs+TIs.

Through LassoCV variable selection (Figure 4), it was observed that a more concise and crucial set of optimal variables was identified, building upon the RF variable selection. Within the variable set of VIs, a total of 10 variables were identified as optimal. In the case of the TI variable set, a total of 23 variables were identified as optimal. Furthermore, in the VI+TI variable set, a total of 26 variables were identified as optimal. As anticipated, the two-step variable selection process (RF-LassoCV) can markedly reduce the variable set without compromising the model’s accuracy, and even enhance the model’s performance.

Table 4 presents the specific optimal variables in different optimal variable sets (including VIs, TIs, and VIs+TIs) after RF-Lasso variable selection. These optimal variables will be utilized as inputs for subsequent machine learning algorithms to develop a superior estimation model for estimating winter wheat SPAD values. This carefully curated set of variables will contribute to improving the estimation accuracy and interpretability of the model, providing accurate estimates of winter wheat growth status.

### 3.3. Model Development and Evaluation

In this research, nine machine learning algorithms, specifically SVR with various kernels (SVR-RBF, SVR-Poly, SVR-Sigmoid, and SVR-Linear), RF, CatBoost, BPNN, LSTM, and ElasticNet, were utilized to estimate SPAD values throughout the entire growth cycle of winter wheat in the sub-study area of Huaisi Town. These machine learning algorithms were applied using different combinations of variables, including VIs and TIs, and incorporating VIs and TIs, refined through RF and LassoCV variable selection.

To optimize the parameters and hyperparameters of the various machine learning algorithms employed in this study, a 5-fold CV combined with grid search was utilized on the training dataset. Table 5 presents the accuracy of different winter wheat SPAD value estimation models on the training dataset (via CV) using various combinations of variables. Each winter wheat SPAD value estimation model was developed using the optimized parameters and hyperparameters, as detailed in Table A3 in Appendix A. The performance of these models was further validated on the training dataset (non-CV) and testing dataset (Table 6).

Regarding the VI variable set, the SVR-Linear model (SVR-Linear_VIs_) demonstrates outstanding estimation performance, with an *R*^2^ of 0.7311, *RMSE* of 3.8778, *RRMSE* of 0.0852, and *RPD* of 1.9531 on the test dataset. In terms of the TI variable set, the SVR-RBF model (SVR-RBF_TIs_) achieves optimal accuracy, with an *R*^2^ of 0.7608, *RMSE* of 3.6578, *RRMSE* of 0.0804, and *RPD* of 2.0706 on the test dataset. In comparison to the SVR-Linear_VIs_ model, this model demonstrated a 4.06% improvement in *R*^2^ and a 5.67% reduction in *RMSE*. The model exhibited commendable performance when modeling with the TI variable set, effectively capturing the dynamic information of SPAD values in winter wheat, thereby enhancing the accuracy of SPAD value estimation.

Furthermore, in the case of the VI+TI variable set, SVR-RBF similarly achieves optimal accuracy, with an *R*^2^ of 0.8131, *RMSE* of 3.2333, *RRMSE* of 0.0710, and *RPD* of 2.3424 on the test dataset. In comparison to the SVR-Linear_VIs_ model, this model demonstrated an 11.22% improvement in *R*^2^ and a 16.627% reduction in *RMSE*. The VI+TI variable set improved the overall accuracy across all models. This promotion further highlights the capability of utilizing multisource data to improve the model’s estimation performance for SPAD values, more comprehensively and accurately reflecting the SPAD values in winter wheat.

Particularly noteworthy is the SVR-RBF_VIs+TIs_ model, which achieved the highest *R*^2^ and *RPD*, as well as the lowest *RMSE* and *RRMSE* on the test set. This performance underscores SVR-RBF_VIs+TIs_ as the optimal cross-growth-stage model capable of estimating SPAD values throughout the entire growth cycle of winter wheat in field environments.

**Figure 5:**
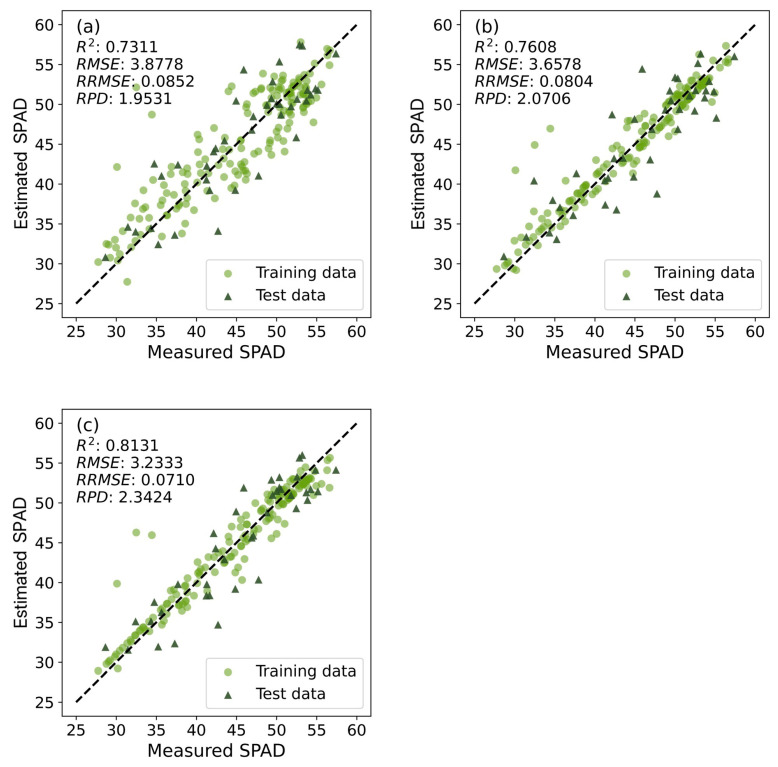
Scatter plots of the optimal models for different variable sets in Huaisi Town. (**a**) SVR-Linear_VIs_; (**b**) SVR-RBF_TIs_; and (**c**) SVR-RBF_VIs+TIs_. Table A3 details the optimal parameter combinations for all models in the scatter plots and the corresponding training dataset accuracies.

For a more in-depth analysis of modeling accuracy, Figure 5 showcases scatter plots comparing the measured SPAD values with those estimated by the top-performing models across the three variable sets (Table 6). The majority of data points in the figure concentrate around the 1:1 diagonal line, signifying the solid agreement between measured and estimated values. This alignment highlights the models’ capability to span diverse growth stages and provide accurate estimations of SPAD values in wheat with minimal discrepancies.

### 3.4. Reliability Verification of the Optimal Model (SVR-RBF_VIs+TIs_)

#### 3.4.1. SHAP Analysis of the Optimal Model

**Figure 6:**
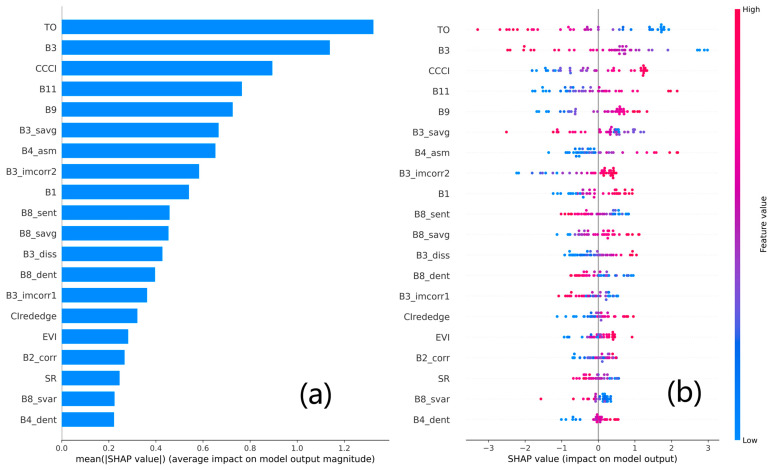
SHAP summary plot for variable importance and impact on SPAD value estimation. (**a**) Variables are ranked vertically according to their impact, from top to bottom; (**b**) Red points indicate that the variable values have a positive impact on the model’s estimation for this observation, while blue points indicate a negative impact. The further a point is from the central line (zero), the greater the variable’s impact on the model’s estimation (positive SHAP values indicate a positive impact, and negative SHAP values indicate a negative impact).

In this study, SHAP values were utilized to further analyze the importance and contribution of variables in the optimal estimation model (SVR-RBF_VIs+TIs_) that was developed based on the Huaisi Town training dataset. Figure 6 integrates variable importance and effect plots, displaying the distribution of SHAP values for the top 20 variables ranked by their influence on the model’s estimation. This visualization aids in understanding the contribution of these variables to the model’s estimation accuracy. Variables at the top of the figure (e.g., TO, B3, CCCI, B11, B9, B3_savg, B4_asm, and B3_imcorr2) exhibit a substantial range of positive (represented by red dots) and negative (represented by blue dots) impacts, indicating significant variability in their effects on model estimations for different SPAD values. Middle-tier variables (e.g., B1, B8_sent, B8_savg, B3_diss, B8_dent, B3_imcorr1, and CIrededge) also show both colors, but with more concentrated distributions, suggesting a relatively smaller impact on model estimations. Variables at the bottom (e.g., EVI, B2_corr, SR, B8_svar, and B4_dent) have the least impact on the model, with most effects close to zero, indicating minimal contribution to model estimations. Overall, both VIs and TIs substantially contribute to the optimal model’s estimation performance. This finding aligns with the observation that incorporating multimodal data consistently enhances the robustness of estimations for winter wheat SPAD values.

#### 3.4.2. Performance Evaluation of the Optimal Model for Winter Wheat with Different Sowing Times in Huaisi Town

**Figure 7:**
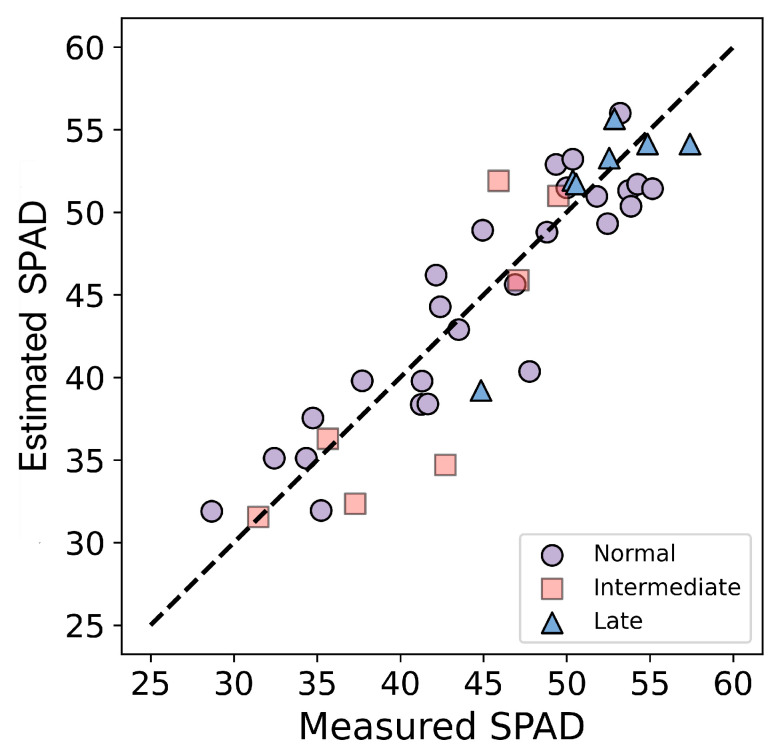
Scatter plot of the optimal model at different sowing times in Huaisi Town.

This research employed the optimal model (SVR-RBF_VIs+TIs_) to validate the SPAD values of field plots at three different sowing times (normal, intermediate, and late) within the testing dataset. These sowing times were determined based on on-site observations. Table 7 shows that the SVR-RBF_VIs+TIs_ model demonstrates favorable performance in estimating SPAD values for different sowing times. The model performs best in the normal sowing time, but also satisfactorily in the intermediate and late sowing time. Additionally, the scatter plot (Figure 7) depicting the measured SPAD values versus estimated SPAD values (test) for winter wheat in different sowing times further illustrates that the optimal model effectively estimates SPAD values across various sowing times.

#### 3.4.3. The Optimal Model’s Transferability in Touqiao Town

This research evaluated the regional transferability of the optimal cross-growth-stage winter wheat SPAD value estimation model (SVR-RBF_VIs+TIs_) by applying it, originally developed from the Huaisi Town training dataset, to Touqiao Town, where sowing times differ significantly.

**Figure 8:**
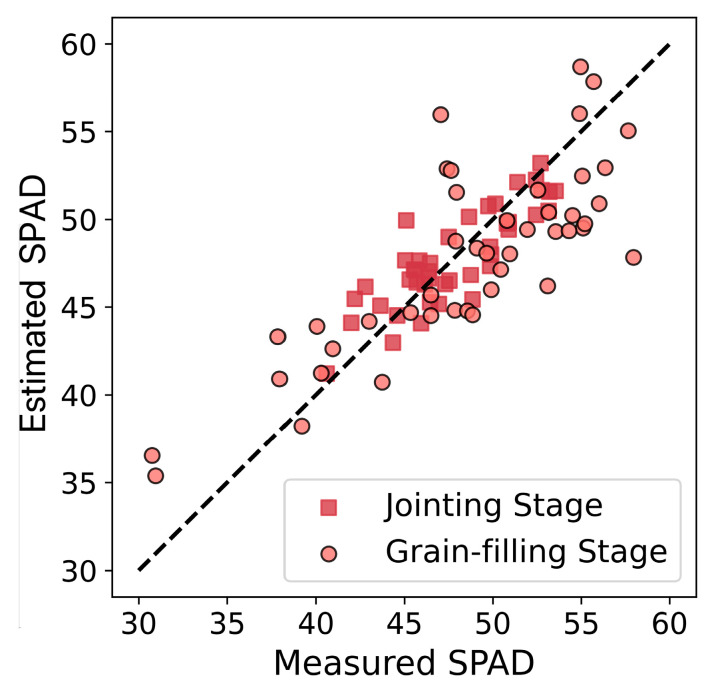
Verification of scatter plots of the optimal model’s transferability in Touqiao Town.

As presented in Figure 8 and Table 8, the results demonstrate that during the jointing stage, the model achieved an *R*^2^ of 0.7091, *RMSE* of 1.8096, and *RRMSE* of 0.0379. During the grain-filling stage, the model achieved an *R*^2^ of 0.6332, *RMSE* of 4.0287, and *RRMSE* of 0.0829. Overall, across both growth stages, the model exhibited an *R*^2^ of 0.6504, *RMSE* of 3.1348, and *RRMSE* of 0.0650. These results indicate that the model possesses favorable transferability in the local region, showcasing its ability to effectively estimate SPAD values in different growth stages and geographical locations.

### 3.5. Impact of the Training Dataset Size on Accuracy

**Figure 9:**
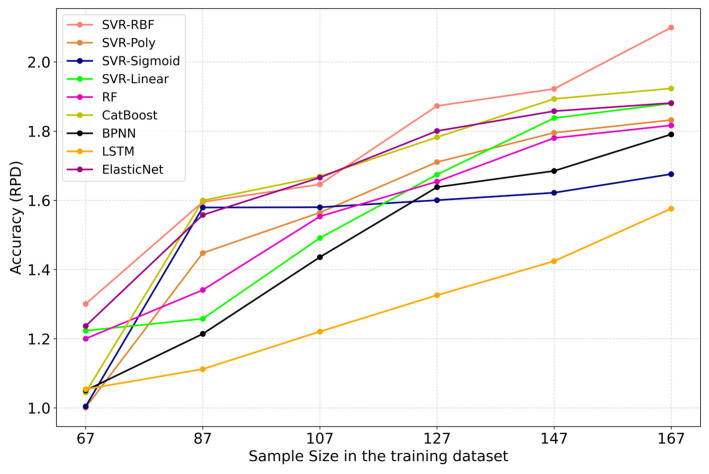
Impact of training dataset size on machine learning algorithms’ performance. To maintain consistency, 5-fold CV was employed during the training phase, followed by testing using the test dataset to evaluate the performance of each machine learning algorithm under varying training dataset sample sizes based on the obtained *RPD* values (the mean *RPD* values obtained for Model_VIs_, Model_TIs_, and Model_VIs+TIs_ represent the *RPD* value for each respective machine learning algorithm). Ranking of different machine learning algorithms based on *RPD* values.

To assess the sensitivity of various machine learning algorithms to different training dataset sample sizes, the training dataset sample sizes were adjusted to 67, 87, 107, 127, 147, and 167 for modeling purposes (including Model_VIs_, Model_TIs_, and Model_VIs+TIs_). The performance of different machine learning algorithms improves as more data are added to the training dataset (Figure 9). Algorithms that exhibit a solid response to adding data include SVR-RBF, SVR-Poly, SVR-Linear, CatBoost, RF, BPNN, LSTM, and ElasticNet. Only SVR-Sigmoid does not show stable performance improvement, highlighting its limited suitability for estimating winter wheat SPAD values. SVR-RBF maintains a consistently high ranking across different training dataset sizes. Additionally, although most machine learning algorithms appear to approach saturation, they may still benefit from even more significant amounts of data to achieve higher accuracy.

## 4. Discussion

### 4.1. The Optimal Model’s Stability and Transferability in the Region

The newly developed optimal model SVR-RBF_VIs+TIs_ for estimating the SPAD values of winter wheat throughout its growth cycle in the region with multiple sowing dates, incorporating VIs and TIs and refined through RF-LassoCV variable selections, utilizing SVR-RBF, has demonstrated superior performance. This model achieved an *RPD* value of 2.3424, exceeding the threshold of 2.0. According to Viscarra Rossel et al. [64], this performance indicates exceptionally good estimation capabilities for estimating the SPAD values of winter wheat throughout its growth cycle. Furthermore, with the highest *R*^2^ value and the lowest *RMSE* and *RRMSE*, this model provides a robust framework for estimating the SPAD values of winter wheat throughout its growth cycle, making it the most outstanding model in the region with multiple sowing dates.

In most research on cereal crops, significant differences in SPAD values at different growth stages have been noted [66]. Additionally, variety and environmental factors (such as soil type, water, and nutrient management, etc.) are significant sources of variation in wheat SPAD values [67,68]. Therefore, the newly developed optimal model’s transferability in the local region was validated in an ‘unknown’ area outside the model’s spatial domain. The area differs in environmental conditions, winter wheat varieties, and sowing times compared to the optimal model’s spatial domain (specific differences are detailed in Section 2.1 (Study Area)). Furthermore, the independent validation dataset includes a growth stage (tillering stage) not covered by the model, which enhances the reliable validation of the optimal model’s transferability in the local region. Therefore, the developed optimal cross-growth-stage model (SVR-RBF_VIs+TIs_), calibrated based on SPAD values under different genotypes and environmental conditions, holds promise for application in other crops or under various environmental conditions in the future.

### 4.2. Impacts of Different Variable Sets on SPAD Value Estimation

Through RF-LassoCV variable selections, various optimal variable sets were generated, minimizing the impact of redundancy and collinearity among the selected variables on subsequent machine learning modeling. As anticipated, the general trend in the estimation performance of the six machine learning algorithms using various optimal variable sets is as follows: VIs+TIs > TIs > VIs.

As reported in previous studies, VIs and other spectral information have become the most commonly used RS indicators in crop parameter monitoring due to their stability and superior performance [59,69]. VIs provide spectral information about the winter wheat canopy. During the development of the SPAD value estimation model for winter wheat in the local region with multiple sowing dates, acceptable estimation outcomes were achieved (optimal model SVR-Linear_VIs_: *R*^2^ = 0.7311, *RMSE* = 3.8778, *RRMSE* = 0.0852, and *RPD* = 1.9531).

It is encouraging to note that this study found that modeling using only TIs extracted from Sentinel-2 MSI’s B2, B3, B4, and B8 bands (with a resolution of 10 m) also demonstrated superior estimation results compared to VIs (optimal model SVR-RBF_TIs_: *R*^2^ = 0.7608, *RMSE* = 3.6578, *RRMSE* = 0.0804, and *RPD* = 2.0706). Results indicate that the TI variable set is a promising alternative to the commonly used VI variable set, particularly for multi-temporal data [70]. This preference may be attributed to the evident variations in the winter wheat canopy structure of fields with different SPAD values observed through satellite images [49,71], significantly contributing to the development of the winter wheat SPAD value estimation model. As described in the study of Colombo et al. [72], TIs were able to show the spatial patterns of crop, shadow, and soil pixels.

Furthermore, many studies based on higher-resolution UAV RS images have reported the potential of incorporating VIs and TIs in monitoring other crop parameters (e.g., nitrogen concentration, yield) [71]. Distinct from previous work, the TIs employed in this study were derived from satellite imagery rather than UAV-based data. In this research, the optimal winter wheat SPAD value estimation model, developed by incorporating VIs and TIs extracted from satellite RS images (SVR-RBF_VIs+TIs_), significantly improved estimation accuracy, irrespective of the machine learning algorithm employed. Incorporating multimodal data consistently provided outstanding performance for the estimation model of winter wheat SPAD values across growth stages.

To date, no studies have been identified that apply satellite-derived TIs for agricultural parameter estimation. In the field of soil RS, only Wang et al. (2025) [73] have recently pioneered the extraction of TIs from different spectral bands of Sentinel-2 MSI (Level-2A) imagery—the same data source adopted in this study—and demonstrated that the integration of spectral and texture information is essential for accurately estimating soil organic carbon content (SOCC). Their findings further underscore the significance of texture information contained in satellite imagery.

While the 10 m resolution of Sentinel-2 MSI imagery is indeed coarser than the scale of individual wheat plants, each pixel still captures aggregate canopy-level variability arising from within-field heterogeneity. Factors such as non-uniform soil nutrient content, slight topographic variations, irrigation patterns, and microclimatic differences result in spatial differences in crop growth, leaf density, and chlorophyll content—even in seemingly homogeneous fields [71,73]. These variations manifest as subtle differences in reflectance and spatial texture on the plot scale.

### 4.3. VIs and TIs in the Optimal Model (SVR-RBF_VIs+TIs_)

Based on the SHAP analysis of the optimal model (SVR-RBF_VIs+TIs_), this study identified several key variables contributing significantly to the estimation of winter wheat SPAD values. The analysis revealed that specific single bands B1, B3, B9, and B11, as well as VIs (such as TO, CCCI, EVI, and SR), contribute significantly to the estimation of winter wheat SPAD values. It is worth noting that the single bands B9 and B11, which contribute significantly to winter wheat SPAD values, both belong to the short-wave infrared (SWIR) region. SWIR wavelengths possess important characteristics associated with nutrients and overall plant physiology, consistent with previous research findings [28]. Additionally, CCCI, EVI, and SR all incorporate two spectral bands, B4 (R, 650–680 nm) and B8 (NIR, 785–900 nm). This principle also parallels the design of the SPAD-502Plus meter, which utilizes two light-emitting diodes (at 650 and 940 nm) and a photodiode detector to measure the transmission of R and NIR light through leaves sequentially [74,75]. The addition of the Rededge (B5, B6, and B7) further enhances the responsiveness of these VIs to winter wheat SPAD values [76].

Existing studies that incorporate TIs to improve crop parameter estimation often lack direct evidence of observable textural variations, even though their objective results underscore the importance of TIs [77]. Some research has attempted to explore the associations between TIs and target variables. For instance, Meng et al. [77] reported that within GLCM-derived TIs, variance and contrast exhibited the strongest correlations with organ biomass, as these two metrics quantify local and global heterogeneity, respectively. Similarly, Yan et al. [78] found that TIs such as R-mean, B-mean, R-diss, R-contrast, G-mean, and R-var were well correlated with SPAD values in pear leaves, whereas TIs derived from the red-edge bands showed relatively poor performance in estimating SPAD values. This finding is largely consistent with the results of this study, in which the optimal GLCM-based TIs—specifically B3_savg, B4_asm, and B3_imcorr2—contributed most significantly to the estimation of winter wheat SPAD values. Specifically, B3_savg reflects the overall brightness and average reflectance level of the winter wheat canopy surfaces captured in Band 3, while B4_asm characterizes the uniformity and regularity of grayscale levels in Band 4, indicating the textural homogeneity of canopy surfaces. Additionally, B3_imcorr2 measures the linear dependency and correlation between pixel pairs at a specified distance in Band 3, providing insights into the spatial structure and arrangement of grayscale levels within the canopy. Changes in structural variables may reflect the growth environment, nutrient status, and physiological condition, which in turn influence the synthesis and content of chlorophyll [22].

### 4.4. Limitations and Future Research

The optimal model developed in this research was only validated within the local region, so its transferability to other regions requires more sample data across larger areas. Collecting additional data will help optimize the transferability of the winter wheat cross-growth-stage SPAD value estimation model developed in this research, which will be a primary focus of our future research.

Furthermore, it is essential to note that this research only covers data collected within one year. The conclusions drawn from the research should be further validated in future studies. This will contribute to ensuring the model’s robustness and reliability, making it better suited for practical applications in different years and environmental conditions.

## 5. Conclusions

By utilizing multi-temporal Sentinel-2 satellite imagery, this research developed a robust model (i.e., SVR-RBF_VIs+TIs_) for estimating winter wheat SPAD values spanning diverse growth stages (from tillering to grain-filling). The model also exhibited good applicability across different sowing times of winter wheat. Furthermore, the model exhibited favorable estimation accuracy at the other sub-study area with different sowing times, wheat varieties, and soil types from the modeling area. Particularly, the jointing stage of winter wheat that was included at the other sub-study area was not included for modeling. These results collectively indicated a demonstrable level of transferability for the estimation model across the region.

This study demonstrated that the incorporation of satellite-derived TIs significantly improved estimation model accuracy compared to the sole use of VIs typical in previous studies. The overall trend in modeling the winter wheat cross-growth-stage SPAD values using different optimal variable sets was as follows: VIs+TIs > TIs > VIs. This result suggested that despite the lower resolution of satellite imagery compared to UAV imagery, the TIs extracted from Sentinel-2 still held promise as a viable alternative to the commonly used VIs.

## Figures and Tables

**Table 1:** An inventory of Sentinel-2 MSI imagery was utilized in this study.

Location	Time	Sentinel-2 MSI Imagery (Level-2A)
Huaisi	17 December 2022	S2B_MSIL2A_20221217T025129_N0509_R132_T50SQB_20221217T051612
	25 February 2023	S2B_MSIL2A_20230225T024709_N0509_R132_T50SQB_20230225T051442
	11 April 2023	S2A_MSIL2A_20230411T024531_N0509_R132_T50SQB_20230411T060701
	13 May 2023	S2B_MSIL2A_20230513T023529_N0509_R089_T50SQB_20230513T054302
Touqiao	8 April 2023	S2A_MSIL2A_20230408T023531_N0509_R089_T50SQA_20230408T071803
	13 May 2023	S2B_MSIL2A_20230513T023529_N0509_R089_T50SQA_20230513T054302

***Note:*** The six Sentinel-2 images are cloud-free over the sampling sites of the study area. Detailed information on the Sentinel-2 spectral bands employed in this study is provided in Appendix A (Table A1).

**Table 2:** RS variables employed in the study.

RS Variables		Formulae	Sentinel-2 MSI Bands	Ref.
VIs	NDVI	*(NIR − R)/(NIR + R)*	B4, B8	[32]
	SCCCI	*(NDVI × RE3)/NDVI*	B4, B7, B8	[33]
	CIrededge	*(NIR/RE3) − 1*	B7, B8	[34]
	IRECI	*NIR − R/(RE2/RE1)*	B4, B5, B6, B8	[35]
	LCI	*(NIR − RE1)/(NIR + R)*	B4, B5, B8	[36]
	CCCI	*[(NIR − RE1)/(NIR + RE1)]/[(NIR − R)/(NIR + R)]*	B4, B5, B8	[33]
	NDVIre	*(NIR − RE1)/(NIR + RE1)*	B5, B8	[37]
	CTVI	*(NDVI + 0.5) × NDVI + 0.5*	B5, B8	[38]
	SIWSI	*(RE4 − SWIR1)/(RE4 + SWIR2)*	B8A, B11, B12	[39]
	mNDVI	*(NIR − SWIR2)/(NIR + SWIR2)*	B8, B12	[40]
	MSI	*SWIR1/NIR*	B8, B11	[41]
	GDVI	*NIR/G*	B3, B8	[42]
	SRT	*SWIR1/SWIR2*	B11, B12	[36]
	AFRI	*NIR − 0.66 × [SWIR1/(NIR + 0.66 × SWIR1)]*	B8, B11	[43]
	SLAVI	*NIR/(RE1 + SWIR2)*	B5, B8, B12	[44]
	OSAVI	*(1 + 0.16) × (NIR − R)/(NIR + R + 0.16)*	B4, B8	[45]
	SR	*NIR/R*	B4, B8	[46]
	DVI	*NIR − R*	B4, B8	[45]
	EVI	*2.5 × (NIR − R)/(NIR + 6 × R − 7.5 × B + 1)*	B2, B4, B8	[47]
	EVI2	*(NIR − R)/(NIR + 2.4 × R + 1)*	B4, B8	[48]
	TCARI	*3 × [(RE1 − R) − 0.2 × (RE1 − G) (RE1/R)]*	B3, B4, B5	[49]
	GCVI	*NIR/G − 1*	B3, B8	[50]
	TO	*TCARI/OSAVI*	B3, B4, B5, B7, B8	[51]
	B1, B2, B3, B4, B5, B6, B7, B8, B8A, B9, B11, B12			/
TIs	asm, contrast, corr, dent, diss, dvar, ent, idm, imcorr1, imcorr2, inertia, savg, sent, svar, var			[52]

***Note:*** Appendix A (Table A2) provides further explanations of GLCM-TIs (asm, contrast, corr, dent, diss, dvar, ent, idm, imcorr1, imcorr2, inertia, savg, sent, svar, and var) extracted using GEE.

**Table 3:** Evaluation of the measured SPAD value data division results.

Location	Data	Period	N	Max	Min	Mean	SD	C·V (%)
Huaisi Town	15 December 2022	Tillering Stage	38	42.70	27.62	33.65	3.81	11.32
	26 February 2023	Green-up Stage	50	50.00	30.10	41.03	4.61	11.25
	9 April 2023	Heading Stage	67	55.60	44.40	51.73	2.35	4.54
	12 May 2023	Grain-filling Stage	54	57.40	32.50	48.54	5.24	10.81
	All		209	57.40	27.62	45.06	7.84	17.41
Touqiao Town	10 April 2023	Jointing Stage	43	53.55	40.60	47.80	3.39	7.10
	14 May 2023	Grain-filling Stage	44	57.95	30.75	48.62	6.73	13.84
	All		87	57.95	30.75	48.21	5.33	11.06

***Note:*** In Huaisi Town, the growth stages presented in the table align with those of winter wheat under conventional sowing schedules, as they represent the predominant cultivation practice within the sub-study area in this research.

**Table 4:** Optimal variables used for subsequent modeling.

Variable	Number	Optimal Variables
VIs	10	IRECI, B9, CCCI, B11, EVI, B1, CIrededge, TO, B3, SR
TIs	23	B8_savg, B2_savg, B4_savg, B2_asm, B3_imcorr2, B8_corr, B8_diss, B8_svar, B8_asm, B4_dvar, B2_dent, B8_imcorr1, B4_svar, B4_imcorr2, B4_ent, B4_corr, B3_corr, B4_sent, B8_idm, B8_ent, B8_sent, B8_dvar, B3_savg
VIs+TIs	26	B4_savg, IRECI, B8_savg, B1, B3_imcorr2, CCCI, B11, EVI, B9, B2_asm, B4_asm, B4_dvar, B8_corr, B4_dent, B8_svar, B3_imcorr1, CIrededge, B8_dent, B2_corr, B3_diss, B3_savg, B8_sent, B2_svar, TO, SR, B3

**Table 5:** Accuracy of SPAD value estimation models (Train (CV)) in Huaisi Town.

Variable	Regression Algorithm		Train (CV)			
			*R* ^2^	*RMSE*	*RRMSE*	*RPD*
VIs	SVR	RBF	0.7932	3.5430	0.0788	2.2056
		Poly	0.6608	4.5379	0.1010	1.7221
		Sigmoid	0.6455	4.6387	0.1032	1.6847
		Linear	0.7497	3.8976	0.0867	2.0050
	RF		0.7411	3.9642	0.0882	1.9713
	CatBoost		0.7420	3.9575	0.0881	1.9746
	BPNN		0.7469	3.7864	0.0843	2.0983
	LSTM		0.7018	4.1578	0.0926	1.8848
	ElasticNet		0.7350	4.0104	0.0892	1.9486
TIs	SVR	RBF	0.7071	4.2167	0.0938	1.8532
		Poly	0.3616	6.2250	0.1385	1.2554
		Sigmoid	0.4136	5.9663	0.1328	1.3098
		Linear	0.6586	4.5522	0.1031	1.7167
	RF		0.6615	4.5331	0.1009	1.7239
	CatBoost		0.7205	4.1186	0.0916	1.8974
	BPNN		0.5377	5.0495	0.1122	1.5779
	LSTM		0.2651	6.3888	0.1420	1.2511
	ElasticNet		0.6705	4.4722	0.0995	1.7474
VIs+TIs	SVR	RBF	0.7564	3.8450	0.0856	2.0324
		Poly	0.6537	4.5847	0.1020	1.7045
		Sigmoid	0.6333	4.7180	0.1050	1.6563
		Linear	0.7664	3.7658	0.0838	2.0751
	RF		0.7374	3.9926	0.0888	1.9573
	CatBoost		0.7563	3.8459	0.0856	2.0319
	BPNN		0.5686	4.7923	0.1065	1.6889
	LSTM		0.4980	5.2065	0.1156	1.5520
	ElasticNet		0.7763	3.6847	0.0820	2.1208

**Table 6:** Accuracy of SPAD value estimation models in Huaisi Town.

Variable	Regression Algorithm		Train (Non-CV)				Test			
			*R* ^2^	*RMSE*	*RRMSE*	*RPD*	*R* ^2^	*RMSE*	*RRMSE*	*RPD*
VIs	SVR	RBF	0.8741	2.7642	0.0615	2.8271	0.7115	4.0166	0.0882	1.8856
		Poly	0.7564	3.8849	0.0856	2.0324	0.6878	4.1787	0.0918	1.8125
		Sigmoid	0.6387	4.6828	0.1042	1.6688	0.6050	4.7004	0.1033	1.6113
		Linear	**0.7717**	**3.7224**	**0.0828**	**2.0994**	**0.7311**	**3.8778**	**0.0852**	**1.9531**
	RF		0.9651	1.4546	0.0324	5.3722	0.6909	4.1580	0.0913	1.8215
	CatBoost		0.9638	1.4832	0.0330	5.2686	0.7149	3.9929	0.0877	1.8968
	BPNN		0.8563	2.9537	0.0657	2.6457	0.6312	4.5416	0.0998	1.6676
	LSTM		0.8730	2.7760	0.0618	2.8150	0.6278	4.5627	0.1002	1.6599
	ElasticNet		0.7520	3.8802	0.0863	2.0140	0.7170	3.9784	0.0874	1.9037
TIs	SVR	RBF	**0.9216**	**2.1821**	**0.0486**	**3.5812**	**0.7608**	**3.6578**	**0.0804**	**2.0706**
		Poly	0.5921	4.9759	0.1107	1.5705	0.5024	5.2756	0.1159	1.4356
		Sigmoid	0.3907	6.0813	0.1353	1.2850	0.6074	4.6856	0.1029	1.6164
		Linear	0.7338	4.0197	0.0894	1.9441	0.6216	4.6005	0.1011	1.6463
	RF		0.9567	1.6208	0.0361	4.8231	0.6601	4.3601	0.0958	1.7371
	CatBoost		0.9614	1.5303	0.0341	5.1066	0.7200	3.9569	0.0869	1.9141
	BPNN		0.9175	2.2379	0.0498	3.4920	0.6804	4.2281	0.0929	1.7913
	LSTM		0.9670	1.4147	0.0315	5.5240	0.4393	5.5996	0.1230	1.3525
	ElasticNet		0.7100	4.1959	0.0934	1.8624	0.6463	4.4479	0.0977	1.7028
VIs+TIs	SVR	RBF	**0.9234**	**2.1564**	**0.0480**	**3.6239**	**0.8131**	**3.2333**	**0.0710**	**2.3424**
		Poly	0.8790	2.7107	0.0603	2.8835	0.7971	3.3683	0.0740	2.2485
		Sigmoid	0.6399	4.6753	0.1040	1.6715	0.6834	4.2081	0.0924	1.7998
		Linear	0.8157	3.3444	0.0744	2.3366	0.7536	3.7124	0.0816	2.0410
	RF		0.9650	1.4576	0.0324	5.3647	0.7133	4.0045	0.0880	1.8913
	CatBoost		0.9638	1.4832	0.0330	5.2686	0.7328	3.8657	0.0849	1.9592
	BPNN		0.9453	1.8225	0.0406	4.2878	0.7198	3.9587	0.0870	1.9132
	LSTM		0.9704	1.3413	0.0298	5.8263	0.6515	4.4147	0.0970	1.7156
	ElasticNet		0.7999	3.4849	0.0775	2.2424	0.7529	3.7178	0.0817	2.0372

***Note:* Bold** indicates the optimal model for each variable set.

**Table 7:** Performance of the optimal model for winter wheat with different sowing dates in Huaisi Town.

Sowing Time	*R* ^2^	*RMSE*	*RRMSE*
Normal	0.8393	3.0024	0.0668
Intermediate	0.5217	4.2848	0.1036
Late	0.4131	2.8105	0.0541

**Table 8:** Validation of the optimal model’s transferability in Touqiao Town.

Location	Period	*R* ^2^	*RMSE*	*RRMSE*
	Jointing Stage	0.7091	1.8096	0.0379
Touqiao	Grain-filling Stage	0.6332	4.0287	0.0829
	All	0.6504	3.1348	0.0650

***Note:*** The jointing stage used to validate the optimal model’s transferability in Touqiao Town was not included in the growth stages that were used to develop the model in Huaisi Town.

## Data Availability

The data are available from the authors upon reasonable request, as the data need further use.

## Appendix A

**Table A1:** Sentinel-2 spectral band information used in this study.

Band Code	Band Name	Sentinel-2A		Sentinel-2B		Resolution (m)
		Central Wavelength (nm)	Bandwidth (nm)	Central Wavelength (nm)	Bandwidth (nm)	
B1	Coastal aerosol	443.9	20	442.3	20	60
B2	Blue	496.6	65	492.1	65	10
B3	Green	560.0	35	559.0	35	10
B4	Red	664.5	30	665.0	30	10
B5	Rededge	703.9	15	703.8	15	20
B6	Rededge	740.2	15	739.1	15	20
B7	Rededge	782.5	20	779.7	20	20
B8	NIR	835.1	115	833.0	115	10
B8A	Narrow NIR	864.8	20	864.0	20	20
B9	Water vapour	945.0	20	943.2	20	60
B11	SWIR	1613.7	90	1610.4	90	20
B12	SWIR	2202.4	180	2185.7	180	20

**Table A2:** The explanation of GLCM-TIs used in this research.

Abbreviation	Full Name	Explanation
asm	Angular Second Moment	Measures the uniformity of pixel values in an image.
corr	Correlation	Measures the linear dependency between pixel intensities at different locations.
dent	Difference Entropy	Measures the randomness or complexity of pixel value differences.
diss	Dissimilarity	Measures the average difference in pixel intensities between neighboring pixels.
dvar	Difference Variance	Measures the variance of pixel intensity differences.
ent	Entropy	Measures the randomness or uncertainty in pixel values.
idm	Inverse Difference Moment	Measures the local homogeneity of pixel values.
imcorr1, imcorr2	Image Correlation 1 and 2	Measures the correlation between different pixel pairs.
inertia	Inertia	Measures the local homogeneity of pixel values.
savg	Sum Average	Measures the distribution of local intensity sums in an image.
sent	Sum Entropy	Measures the randomness or complexity of pixel intensity sums.
svar	Sum Variance	Measures the variance of local intensity sums in an image.
var	Variance	Measures the variance of pixel intensities in an image.

**Table A3:** Accuracy of SVR models with different kernels and optimal parameters.

Variable	Model	C	Epsilon
VIs	SVR-RBF	17.48	1.00
	SVR-Poly	7.65	0.42
	SVR-Sigmoid	0.91	0.01
	SVR-Linear	1.00	0.20
TIs	SVR-RBF	29.27	1.00
	SVR-Poly	15.27	4.48
	SVR-Sigmoid	1.27	0.01
	SVR-Linear	1.00	0.90
VIs+TIs	SVR-RBF	19.94	1.00
	SVR-Poly	17.05	0.46
	SVR-Sigmoid	1.03	0.01
	SVR-Linear	0.60	0.60
